# Cancer screening recommendations: an international comparison of high income countries

**DOI:** 10.1186/s40985-018-0080-0

**Published:** 2018-03-02

**Authors:** Mark H. Ebell, Thuy Nhu Thai, Kyle J. Royalty

**Affiliations:** 1 College of Public Health, University of Georgia, 125 Miller Hall, UGA Health Sciences, GA 30602 Athens, Georgia USA; 2 Department of Pharmaceutical Outcomes and Policy, College of Pharmacy, University of Florida, Gainesville, Florida USA; 3 Augusta University/University of Georgia Medical Partnership Campus, Athens, Georgia USA

**Keywords:** Cancer screening, Breast cancer, Colorectal cancer, Prostate cancer, Cervical cancer, Overdiagnosis, Healthcare economics, Lung cancer, Skin cancer

## Abstract

**Background:**

Recommendations regarding cancer screening vary from country to country, and may also vary within countries depending on the organization making the recommendations. The goal of this study was to summarize the cancer screening recommendations from the 21 countries with the highest per capita spending on healthcare.

**Main body:**

Cancer screening guidelines were identified for each country based on a review of the medical literature, internet searches, and contact with key informants in most countries. The highest level recommendation was identified for each country, in the order of national recommendation, cancer society recommendation, or medical specialty society recommendation. Breast cancer screening recommendations were generally consistent across countries, most commonly recommending mammography biennially from ages 50 to 69 or 70 years. In the USA, specialty societies generally offered more intensive screening recommendations. All countries also recommend cervical cancer screening, although there is some heterogeneity regarding the test (cytology or HPV or both) and the age of initiation and screening interval. Most countries recommend colorectal cancer screening using fecal immunochemical (FIT) testing, while only seven countries recommend general or selective screening for prostate cancer, and a similar number explicitly recommend against screening for prostate cancer. Screening for lung and skin cancer is only recommended by a few countries. Greater per capita healthcare expenditures are not associated with greater screening intensity, with the possible exception of prostate cancer.

**Conclusions:**

Guidelines for cancer screening differ between countries, with areas of commonality but also clear differences. Recommendations have important commonalities for well-established cancer screening programs such as breast and cervical cancer, with greater variation between countries regarding prostate, colorectal, lung, and skin cancer screening. Ideally, recommendations should be made by a professionally diverse, independent panel of experts that make evidence-based recommendations regarding screening based on the benefits, harms, and available resources in that country’s context.

## Background

Cancer is the second leading cause of death worldwide [1], with 8.8 million cancer deaths in 2015 [1], and over 14 million new cancer cases diagnosed in 2012 [2]. About 30% of cancer deaths occurred in high-income countries [1]. Cancer screening programs have the potential to reduce cancer-specific and possibly all-cause mortality [3, 4]. The United States Preventive Services Task Force (USPSTF) has concluded that there is at least adequate evidence of a net benefit for screening for lung, breast, cervical, and colorectal cancers [5–8], and screening programs for cancer are widespread in other high resource countries [3, 4, 9–11]. For example, of over 31 million women eligible for breast cancer screening in European countries, 79% were invited to screen and 49% were screened [3]. Breast cancer mortality has decreased in the USA from 31.4 to 20.5 deaths per 100,000 women between 1975 and 2014, with similar trends in Europe, an effect attributable to both screening and improved treatment [12].

Cancer screening programs have largely been implemented in high-income countries with greater available resources [9, 13, 14]. However, there is considerable variation in terms of screening methods, starting age, stopping age, and screening interval between countries [3, 4, 9]. For example, the USPSTF recommends that adults aged 50 to 75 years should be screened for colorectal cancer with one of seven tests or combinations of tests (including colonoscopy), [8] while the Canadian Task Force on Preventive Health Care does not recommend colonoscopy as a screening test [15]. In Europe, even after the European Union recommendations in 2003, the implementation of the Council recommendations differs between countries [3]. We hypothesize that countries spending more on healthcare per capita will have more intensive cancer screening recommendations, screening for more cancers over a longer age range, and at a shorter screening interval (Appendix).

In the current report, we will review and compare cancer screening recommendations implemented in 21 high resource countries that in 2015 spent at least $3000 per capita on healthcare. We will review national recommendations where available, or the most relevant other guidelines for that country where national or federal recommendations such as those of the USPSTF do not exist. The cancers addressed will include breast, cervical, colorectal, prostate, skin, and lung cancer. As a point of comparison with US national guidelines from the USPSTF, we include relevant specialty society guidelines from the American College of Radiology, American College of Obstetrics and Gynecology, and others.

## Methods

The goal of the study was to compare cancer screening recommendations in countries with comparable levels of healthcare spending, and to try to understand the relationship between healthcare spending and the intensity of screening recommendations. Membership in the Organization for Economic Cooperation and Development (OECD) was chosen as the initial qualification for inclusion in the study to ensure that the countries compared were similar economically. It was then decided that total health expenditure per capita was the most relevant statistic to national cancer screening recommendations. Therefore, the 35 OECD member countries were rank ordered according to total healthcare expenditure per capita using data from 2015. From this ranked list, all countries that spent over USD3000 per capita on health that year were included (*n* = 21) [16].

The next step was to determine which national organization’s cancer screening guidelines would be chosen as that country’s national recommendations. First, colleagues of the authors in each country of the study were consulted as local content experts. This was supplemented by searches of the internet where necessary. Once the national recommendations were determined for each country, the data were abstracted in tandem by two members of the research team, consulting with the project leader (Dr. Ebell) to resolve any discrepancies.

To create a uniform grading scheme, each screening test for each type of cancer was graded as *recommended* (which includes both strongly recommended and recommended*)*, *recommended selectively*, *recommended against*, or *insufficient evidence*, corresponding to the categories used by the US Preventive Services Task Force. After analysis of each country’s national screening recommendations, the data were organized into tables. Finally, screening intensity was defined for breast, cervical, and colorectal cancer as the total number of lifetime screening tests recommended for an average risk person.

## Results

Information for the organizations making screening recommendations is shown in Table 1. We abstracted the screening’s recommendations from national guideline committee’s websites for 15 out of the 21 selected countries. The other countries’ recommendations are from cancer society’s websites. Notably, for the USA, beside the United Stated Preventive Services Task Force (USPSTF), we additionally abstracted the recommendations from the American Cancer Society (since it is widely used by US physicians) and three specialty society’s websites.

**Table 1:** Organizations making screening recommendations, by country

Country	Organization Name	Type of organization
United States	United States Preventive Services Task Force	National guideline committee
United States	American Cancer Society	Cancer society
United States	American College of Obstetrics & Gynecology	Specialty society
United States	American College of Radiology	Specialty society
United States	American Urological Association	Specialty society
Luxembourg	Ministry of Health	National guideline committee
Switzerland	League Against Cancer	Cancer society
Norway	Cancer Registry of Norway	Cancer society
Netherlands	National Institute for Public Health and the Environment	National guideline committee
Germany	Federal Joint Committee	National guideline committee
Sweden	National Board of Health and Welfare	National guideline committee
Ireland	National Screening Service	National guideline committee
Austria	Austrian Cancer Aid Society	Cancer society
Denmark	National Board of Health	National guideline committee
Belgium	Foundation Against Cancer	Cancer society
Canada	Canadian Task Force for Preventive Health Care	National guideline committee
Australia	Australian Government Department of Health	National guideline committee
France	National Cancer Institute	National guideline committee
Japan	National Cancer Center	National guideline committee
Iceland	Icelandic Cancer Society	Cancer society
United Kingdom	United Kingdom National Screening Committee	National guideline committee
Finland	Cancer Society of Finland	Cancer society
New Zealand	Ministry of Health	National guideline committee
Italy	National Screening Observatory	National guideline committee
Spain	Cancer Strategy of National Health System	National guideline committee

### Breast cancer screening

The recommendations for breast cancer screening with mammography are presented in Table 2. Overall, the recommendations for breast cancer are quite similar among the 21 selected countries. The most common screening age ranges are from 50 to 69 years old, and most of those countries are European. Screening for breast cancer every 2 years is recommended in most of the countries. Only Japan does not specify the screening interval. The American College of Radiology has the longest age range for screening and is the only guideline in the world recommending an annual screening interval, while the United Kingdom has the longest screening interval (every 3 years).

**Table 2:**
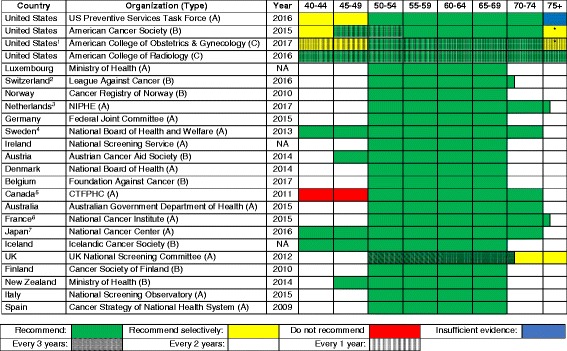
Recommendations for breast cancer screening with mammography, in order of overall healthcare spending

Type of organization: *A* national guideline committee, *B* cancer society, *C* specialty society, *D* other

^1^United States American College of Obstetrics and Gynecology: screening interval of 1 or 2 years

^2^Switzerland: screening age of 50–70 years

^3^Netherlands: screening age of 50–75 years

^4^Sweden: screening interval is 18 months from age 40 to 54 years

^5^Canada: screening interval of 2 or 3 years

^6^France: screening age of 50–75 years

^7^Japan: Do not recommend a screening interval

Abbreviations: *UK* United Kingdom, *USA* United States of America, *NIPHE* National Institute for Public Health and the Environment, *CTFPHC* Canadian Task Force on Preventive Health Care, *NA* not available (cannot find information)

*Consider the person’s life expectancy when making a decision

### Cervical cancer screening

Table 3 describes cervical cancer screening’s recommendations. Luxembourg is the only country with no national recommendation identified for cervical cancer screening. There was some heterogeneity regarding the recommended tests (cytology, HPV, or both), the age to begin screening, and screening intervals. Most countries recommend an age of initiation of screening from 18 to 29 years and a stopping age between 60 and 70 years. Countries most commonly recommend a screening interval of 3 to 5 years. Six out of the 21 countries have adopted HPV testing as a primary test for cervical cancer screening, and cytology is still predominantly recommended with a screening interval of 3 years.

**Table 3:**
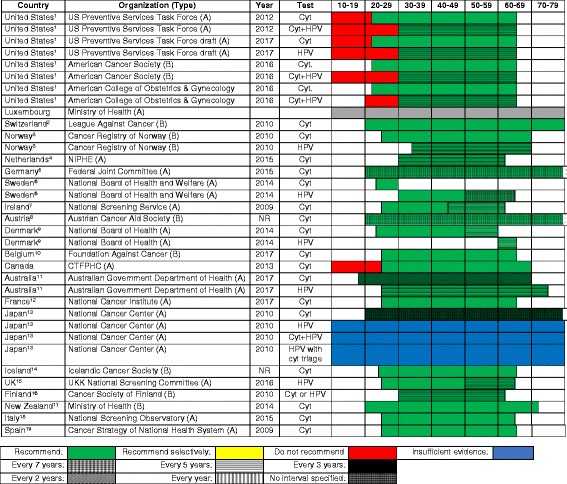
Recommendations for cervical cancer screening, in order of overall healthcare spending

Type of organization: *A* national guideline committee, *B* cancer society, *C* specialty society, *D* other

Start and stop age coding: *wavy border*—not specified

Most guidelines recommend that screening cease between ages 65 and 70 years in women with consistently normal screening in the previous decade. Screening is not recommended for women who have had a hysterectomy for benign disease

^1^USA: screening age of 21–65 years for cytology and 30–65 years for cytology and HPV testing

^2^Switzerland: start and stop ages are not recommended

^3^Norway: screening age of and 25–69 years for cytology and 34–69 years for HPV testing

^4^Netherlands: screening age of 30–60 years

^5^Germany: stop age is not recommended

^6^Sweden: screening age of 23–29 years for cytology and 30–64 years for HPV testing

^7^Ireland: screening age of 25–61 years

^8^Austria: stop age is not recommended

^9^Denmark: screening age of 23–59 years for cytology and 60–64 years for HPV testing

^10^Belgium: screening age of 25–65 years for cytology

^11^Australia: screening age of 18–69 years for cytology (current recommendation) and 25–75 years for HPV testing (will implement from December 2017)

^12^France: screening age of 25–65 years

^13^Japan: stop age and screening interval are not recommended

^14^Iceland: screening age of 23–65 years

^15^UK: screening age of 25–64 years

^16^Finland: screening age of 30–60 years

^17^New Zealand: screening age of 20–70 years

^18^Italy: screening age of 25–64 years (some programs have moved into 25–30/35 years with cytology and 30/35–64 years with HPV testing)

^19^Spain: screening age of 25–65 years

Abbreviations: *USPSTF* United States Preventive Services Task Force, *NIPHE* National Institute for Public Health and the Environment, *CTFPHC* Canadian Task Force on Preventive Health Care, *NA* not applicable (cannot find information), *Cyt.* cytology, *Cyt + HPV* cytology plus HPV co-testing

+Date website with recommendation last updated

### Colorectal cancer screening

Table 4 demonstrates the variability in screening recommendations across different countries with respect to both the type and schedule of screening. Even within the USA differences in guidelines exist, as the USPSTF and American Cancer Society (ACS) express no preference concerning the type of test, while the recent joint recommendations from several specialty societies recommend a tiered approach with colonoscopy or fecal immunochemical (FIT) testing offered first [17]. All other countries recommend a test for fecal occult blood, colonoscopy, or either. Regarding fecal occult blood testing, FIT is generally preferred over gFOBT, especially in more recent guidelines. Concerning the use of colonoscopy as a screening method, the only countries outside the USA that recommend it are Switzerland, Germany, and Austria. In some countries, the infrastructure may be lacking in terms of the number of trained gastroenterologists to support this screening method, although cost and acceptability are also important factors. The results summarized in Table 4 reflect no clear association between total healthcare expenditure per capita and colorectal cancer screening recommendations, other than that 4 of 5 countries recommending the shortest screening interval are among the lowest spenders per capita. Notably, only Austria and Japan recommend initiation of screening for average risk persons at age 40.

**Table 4:**
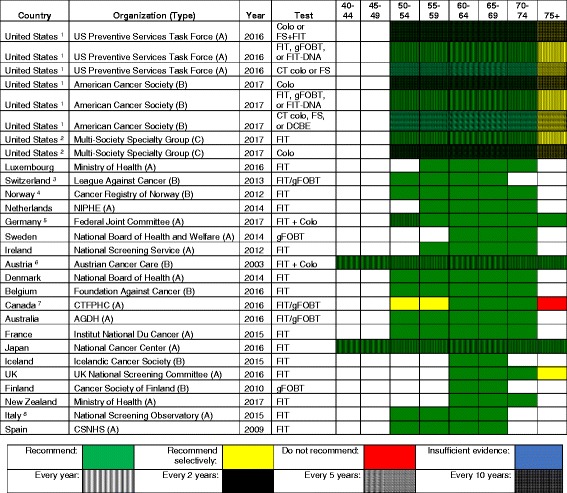
International colorectal cancer screening recommendations for the general population (persons not at “high-risk”), in order of decreasing total healthcare expenditure per capita

Type of organization: *A* national guideline committee, *B* cancer society, *C* specialty society or societies

^1^USPSTF and ACS: no preference regarding choice of tests; high sensitivity gFOBT preferred, with recommendation against in-office FIT or gFOBT; FIT-DNA is every 1 or 3 years; FS + FIT is FS every 10 years and FIT annually

^2^US Multi-Society Guidelines: consider screening between 75 and 85 if no previous screening

^3^Switzerland: or colonoscopy every 10 years

^4^Norway: currently being compared to flexible sigmoidoscopy (with screening once every 10 years from age 50 to 74) in a pilot study

^5^Germany: beginning at age 55, also obtain colonoscopy every 10 years (at a minimum), stopping after 2 colonoscopies

^6^Austria: beginning at age 50, also obtain colonoscopy every 7–10 years

^7^Canada: or flexible sigmoidoscopy every 10 years; colonoscopy is not recommended

^8^Italy: FIT or flexible sigmoidoscopy once at age 58 or 60, and then FIT every 2 years from age 59 to 69

*Abbreviations: USA* United States of America, *Colo* colonoscopy, *CT colo* CT colonography, *FIT* fecal immunochemical test, *gFOBT* guaiac fecal occult blood test, *NIPHE* National Institute for Public Health and the Environment, *CTFPHC* Canadian Task Force on Preventive Health Care, *CSNHS* Cancer Strategy of the National Health System, *AGDH* Australian Government Dept of Health

### Prostate cancer screening

Table 5 demonstrates the variability between various countries regarding screening for prostate cancer. Unlike that with the other cancers, the chief disagreement concerning prostate cancer is not between the type and frequency of test but rather whether or not to screen at all. In fact, if one includes the current 2012 USPSTF recommendation, eight countries explicitly recommend against prostate cancer screening. Of the remaining countries that did not recommend against screening, the vast majority did not have an organized national screening program in place, recommended that an individual consult with their physician or did not make a recommendation.

**Table 5:**
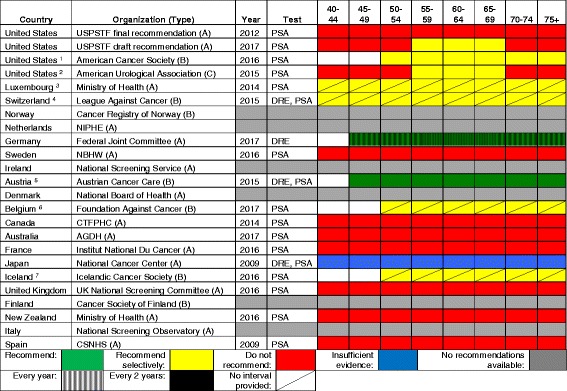
International prostate cancer screening recommendations for the general population (persons not at “high-risk”), in order of decreasing total healthcare expenditure per capita

Interval coding: *vertical cross-hatch* every year, *solid* every 2 years, *diagonal line* no interval provided

Type of organization: *A* national guideline committee, *B* cancer society, *C* specialty society

^1^US American Cancer Society: screening should be done every year if PSA levels are greater than 2.5 ng/mL; stop screening in asymptomatic men with less than 10 years life expectancy; men at increased risk should consider beginning screening at 40 or 45 years

^2^US American Urological Association: an interval of 2 years or more is preferred. Additionally, baseline PSA levels can be used to individualize rescreening intervals. Screening should be stopped in asymptomatic men whose life expectancy is less than 10 years

^3^Luxembourg: age to start screening not provided. No organized national guidelines; screening based on individual discussion between patient and physician

^4^Switzerland: age to start screening not provided. No organized national guidelines; screening based on individual discussion between patient and physician

^5^Austria: “Screening should be carried out ‘regularly’ from the 45th birthday”

^6^Belgium: no organized national guidelines; screening based on individual discussion between patient and physician

^7^Iceland: no organized national guidelines; screening based on individual discussion between patient and physician

Abbreviations: *USPSTF* United States Preventive Services Task Force, *PSA* prostate-specific antigen, *NIPHE* National Institute for Public Health and the Environment, *DRE* digital rectal examination, *NBHW* National Board of Health and Welfare, *CTFPHC* Canadian Task Force on Preventive Health Care, *AGDH* Australian Government Department of Health, *CSNHS* Cancer Strategy of the National Health System

### Skin cancer screening

Most countries included did not make a recommendation regarding skin cancer screening. Only the USA, Germany, Austria, and France address skin cancer screening. The latter three recommend screening, while the USPSTF has determined that there is insufficient evidence to recommend visual skin examination by a physician. Germany recommends that such examination take place every 2 years beginning at age 35, and France provides seven questions for general practitioners to ask their patients in order to assess risk. Uniquely, Austria recommends self-examination and recommends doing so twice a year (before and after the summer months). None of the countries recommending screening provide an age to stop screening.

### Lung cancer screening

Among 21 selected countries, only 5 countries have recommendations regarding lung cancer screening (USA, Canada, Japan, United Kingdom, and Australia). Australia and the United Kingdom recommend against lung cancer screening. Both the USPSTF and the Canadian Task Force recommend low-dose computed tomography (CT) for smokers with at least 30 pack-year smoking history and who smoke or quit smoking less than 15 years. However, the USPSTF recommends screening for the age range 55 to 80 years, while the Canadian Task Force recommends screening for a narrower age range from 55 to 74 years, with a screening interval of 1 to 3 years. In contrast to the USA and Canada, Japan recommends chest radiography to screen for lung cancer starting at age 40 years, but concludes that there is insufficient evidence for low-dose CT. Japan does not recommend a stopping age or screening interval. Several European countries have screening trials underway, so these recommendations may change when those results are available [18, 19].

### Association between healthcare expenditures and screening intensity

**Fig. 1:**
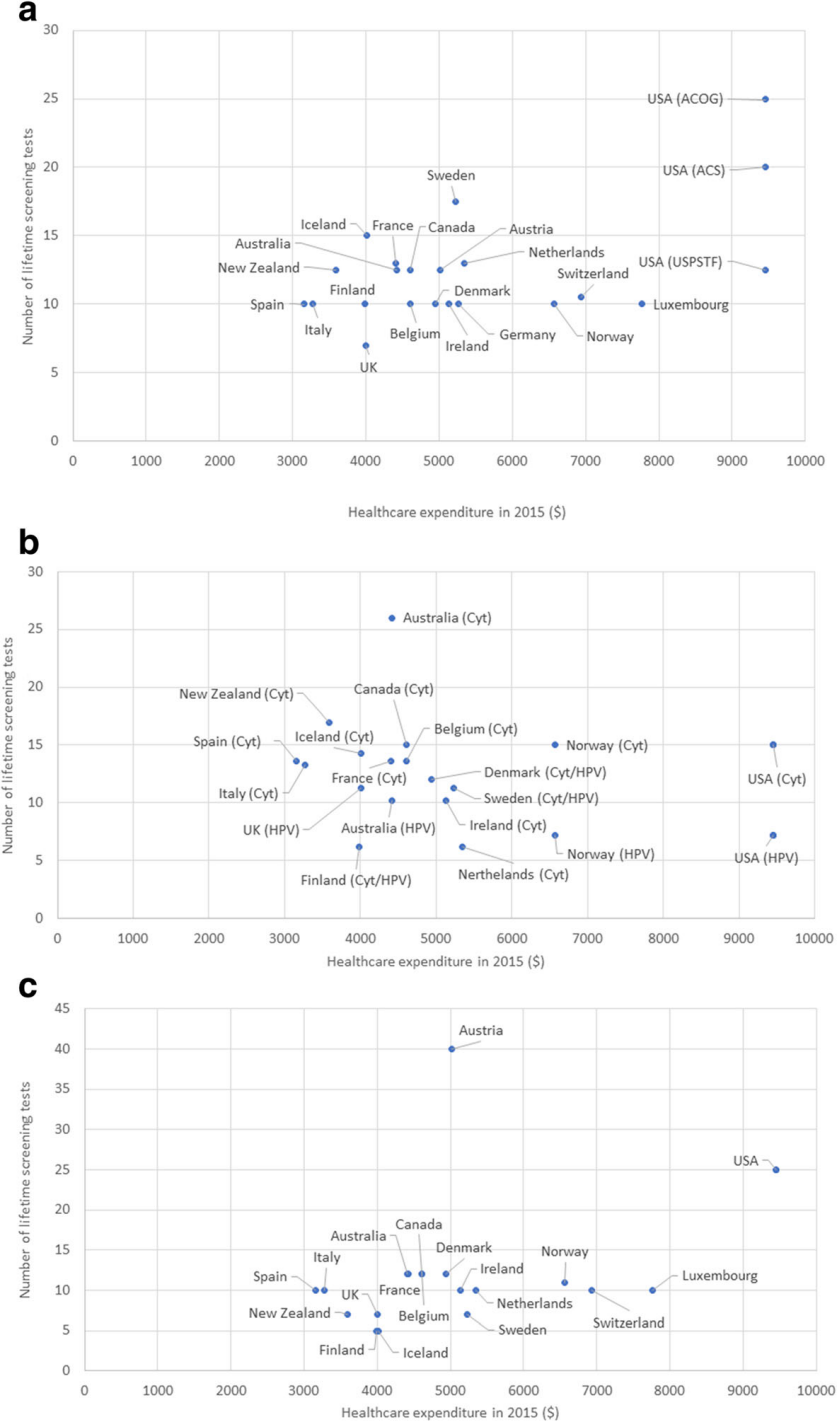
This figure shows the relationship between per capita spending on healthcare with the number of lifetime screening tests recommended for **a**) breast cancer (mammography), **b**) cervical cancer (cytology or HPV), and **c**) colorectal cancer (FIT)

Figure 1 summarizes the relationship between healthcare expenditures and the intensity of screening, measured as the total lifetime number of screening tests recommended for an average risk person for breast, cervical, and colorectal cancer (the latter based on recommendations for use of the FIT). It shows that there is no clear association between expenditures and screening intensity for these cancers, disproving our initial hypothesis. Regarding prostate cancer, 5 of the top 10 countries in terms of healthcare expenditure recommend selective or routine screening, compared with only 2 of the bottom 10 countries. Finally, the three countries recommending lung cancer screening are ranked 1st, 12th, and 15th in spending, again creating no pattern of higher spending equating to higher intensity of screening.

## Discussion

We have summarized national screening recommendations for the 21 countries with the highest total healthcare expenditure per capita. These countries were selected because they are likely to have adequate resources for a cancer screening program of some kind; comparison with less well-resourced settings would be unfair and would be less likely to reflect the differences in values, priorities, and the assessment of evidence that we are interested in. As physicians and researchers in the USA, we are especially interested in how our recommendations compare with other countries, and what we can learn from them.

In comparing recommendations, we are not making value judgements or endorsing a single “correct” answer regarding age at initiation, screening interval, or age at cessation of screening. These decisions likely reflect differences between countries and organizations regarding how benefits and harms are valued and balanced at a societal level, the type of evidence considered, the availability of resources and infrastructure for screening (for example, adequate trained medical personnel to perform colonoscopy), whether and how cost is considered, and different standards for assembling, evaluating, and interpreting a complex evidence base. For example, does the body making a recommendation consider only randomized controlled trials with cancer-specific mortality as the outcome, or does it also consider observational data and modeling studies? [20] Does the panel consider outcomes beyond mortality, such as disease progression, cost, stage shifts, or quality of life? Is cost explicitly considered or is the decision made solely on the balance of potential benefits and harms?

Note that in our discussion below, we consider the USPSTF to represent the US national screening recommendation, although recommendations from USA specialty and cancer societies are also presented. Also, when we use the term “more aggressive” or “more conservative” to describe screening recommendations, we refer to recommendations with a broader age range and/or a more frequent interval vs a narrower age range and/or a less frequent interval. Finally, a review of the process used by each country or organization to develop and update guidelines is beyond the scope of this article.

### Commonalities

There was considerable homogeneity regarding screening recommendations for breast cancer, cervical cancer, and to some extent, colorectal cancer. A starting age for routine mammography of 50 years was recommended by 16 of 21 countries; all countries but one recommended a biennial interval, and 14 recommended a stopping age of 69 or 70 years. Similarly, most countries recommended cervical cancer screening beginning between 21 and 30 years of age (depending on whether or not testing for human papillomavirus was employed), and most recommended a stopping age between 65 and 70 years. Similarly, most countries recommended that colorectal cancer screening begin at age 50 or 55 years and stop by 75 years. Finally, there is a general consensus against screening for lung cancer and melanoma, with only the USA, Japan, and Canada recommending lung cancer screening and only Germany, Austria, and France recommend some approach to screening for skin cancer.

### Differences

The approach to screening for cervical cancer is evolving, with some countries still recommending cytology only, some recommending HPV testing or co-testing, and some giving clinicians the option of choosing the favored approach. Four countries (Iceland, UK, Sweden, and Finland) do not recommend screening for colorectal cancer until age 60, compared to start ages between 40 and 50 years for most other countries. The recommended test for colorectal cancer screening also varies. FIT was most widely recommended, while the USPSTF offered seven different options, and Germany and Austria recommended FIT for younger patients followed by a series of colonoscopies. There is considerable heterogeneity regarding prostate cancer screening: seven countries recommend screening for prostate cancer in some form, while eight explicitly recommend against it. This is likely due to variation in how guideline panels assess the potential benefits and harms.

### Variation by country and type of organization

For prostate cancer screening, of the seven countries with a recommendation to screen or screen selectively for prostate cancer, five were from the top half of the selected countries based on per capita health expenditures. Four of the five countries with the shortest interval for colorectal cancer screening (Iceland, UK, Finland, and New Zealand) are among the six lowest spending countries. However, there was no apparent association between per capita health expenditures and the intensity of screening for breast and cervical cancer.

Other than within the USA, there was no clear difference in terms of the intensity of screening recommendations coming from national guideline committees, cancer societies or leagues, and specialty societies. In the USA, the recommendations regarding mammography from the American Cancer Society, American College of Obstetrics and Gynecology, and American College of Radiology were the only ones identified that recommended annual screening and also had longer screening intervals for patients at average risk. The American College of Radiology was the only body that recommended annual mammography starting at age 40 years, with no specified stopping age. On the other hand, there is considerable similarity regarding colorectal cancer screening between the USPSTF, ACS, and specialty society guidelines.

Prostate cancer screening recommendations are now similar between the USPSTF draft recommendation of 2017 and the American Urology Association, and recommendations regarding cervical cancer screening between the USPSTF, ACS, and ACOG are nearly identical. Assuming that they are based on the best available evidence, this kind of “harmonization” between guidelines from different groups within a country sends a clear, unified message to patients and physicians. In the absence of such harmonization, confusion may reign and physicians may do what feels right or what is requested by patients rather than what is supported by the best evidence. Due to medicolegal concerns, some physicians may feel compelled to practice based on the most aggressive set of recommendations or based on patient request. This is especially true in the USA context, where “failure to diagnose” is the most common reason for a malpractice lawsuit [21].

## Conclusions

Guidelines for cancer screening differ between countries, with areas of commonality but also clear differences. Per capita healthcare spending among wealthy countries appears to have relatively little impact on recommendations; differences are more likely to stem from variation in how benefit and harm are evaluated, and which evidence is considered. Intellectual and financial conflicts of interest inherent to professional societies may contribute to more aggressive recommendations for breast cancer screening from these groups in the USA. A greater intensity of screening may alter the balance of benefits and harms by increasing the likelihood of direct harms of the screening test, as well as the serious harms associated with overdiagnosis [22]. In some cases, where specialty societies have adopted more rigorous methods, their recommendations have become more conservative, as when the American Urologic Association moved from a consensus to an evidence-based process [23]. Of course, one could also argue that national bodies like the USPSTF or Canadian Task Force are overly conservative since they rely largely on randomized trials and modeling for their recommendations. And, even groups using similarly rigorous methods may reach different conclusions, with USA and Canadian recommendations for colorectal cancer screening a good example.

In conclusion, we encourage the formation of independent panels of experts in each country, modeled after the USPSTF, Canadian Task Force, and the UK National Screening Committee. These panels should make independent, evidence-based recommendations regarding screening that assess the benefits, harms, and available resources in that country’s context. Consistent with IOM recommendations, the panels should include primary care physicians, patients, methodologic experts and relevant subspecialists, recommendations should be regularly updated, and panel members should be free of financial and intellectual conflict of interest to the greatest extent possible [24].

## Appendix

**Table 6:** The references of the national recommendations for breast, cervical, colorectal, prostate, skin, and lung cancer screening by country

Country	Reference	Year of recommendation or most recent update
Breast cancer		
United States (USPSTF)	https://www.uspreventiveservicestaskforce.org/Page/Document/UpdateSummaryFinal/breast-cancer-screening1	2016
United States (ACS)	https://www.cancer.org/cancer/breast-cancer/screening-tests-and-early-detection/american-cancer-society-recommendations-for-the-early-detection-of-breast-cancer.html	2015
United States (ACOG)	http://www.acog.org/About-ACOG/News-Room/Practice-Advisories/Practice-Advisory-Breast-Cancer-Screening	2017
United States (ACR)	https://www.acr.org/Advocacy-and-Economics/ACR-Position-Statements/Breast-Cancer-Screening-for-Average-Risk-Women	2017
Luxembourg (Ministry of Health)	https://plancancer.lu/about/depistage/cancer-du-sein/depistage-actuel-cancer-du-sein/	NA
Switzerland (League Against Cancer)	https://www.liguecancer.ch/prevenir-le-cancer/depistage-et-prevention-du-cancer/cancer-du-sein/depistage-par-mammographie/ https://www.swisscancerscreening.ch/fr/cancer-du-sein/informations-generales/mammographie	2016
Norway (Cancer Registry of Norway)	https://www.kreftregisteret.no/en/screening/Breast-Cancer-Screening-Programme	2010
Netherlands (NIPHE)	http://www.rivm.nl/en/Topics/B/Breast_cancer_screening_programme	2017
Germany (Federal Joint Committee)	https://www.g-ba.de/institution/themenschwerpunkte/frueherkennung/krebsfrueherkennung/	2015
Sweden (National Board of Health and Welfare)	http://www.socialstyrelsen.se/riktlinjer/nationellascreeningprogram/brostcancer-screeningmedmammog	2013
Ireland (National Screening Service)	http://www.screeningservice.ie/screening.html	NA
Austria (Austrian Cancer Aid Society)	https://www.krebshilfe.net/information/krebs-vorsorge/mammografie-screening/ https://ec.europa.eu/health/sites/health/files/major_chronic_diseases/docs/2017_cancerscreening_2ndreportimplementation_en.pdf	2014
Denmark (National Board of Health)	https://www.sst.dk/da/udgivelser/2014/~/media/31CB15B2A39E4A3EB198B9486F66B4A2.ashx	2014
Belgium (Foundation Against Cancer)	http://www.cancer.be/les-cancers/types-de-cancers/cancer-du-sein/examens	2017
Canada (CTFPHC)	http://canadiantaskforce.ca/guidelines/published-guidelines/breast-cancer/	2011
Australia (Australian Government Department of Health)	http://www.cancerscreening.gov.au/internet/screening/publishing.nsf/Content/breast-screening-1	2015
France (National Cancer Institute)	http://www.e-cancer.fr/Professionnels-de-sante/Depistage-et-detection-precoce/Depistage-du-cancer-du-sein/Orienter-vos-patientes	2015
Japan (National Cancer Center)	http://canscreen.ncc.go.jp/guideline/nyugan.html	2016
Iceland (Icelandic Cancer Society)	https://www.krabb.is/leitarstod/um-leitarstodina/af-hverju-brjostakrabbameinsleit/leit-ad-krabbameini-i-brjostum-1	NA
UK (UK National Screening Committee)	https://legacyscreening.phe.org.uk/breastcancer http://www.nhs.uk/Conditions/breast-cancer-screening/Pages/When-its-offered.aspx	2012
Finland (Cancer Society of Finland)	https://www.cancer.fi/syoparekisteri/en/mass-screening-registry/breast_cancer_screening/screening_programme/ https://ec.europa.eu/health/sites/health/files/major_chronic_diseases/docs/2017_cancerscreening_2ndreportimplementation_en.pdf	2010
New Zealand (Ministry of Health)	https://www.nsu.govt.nz/breastscreen-aotearoa/what-breast-cancer	2014
Italy (National Screening Observatory)	http://www.osservatorionazionalescreening.it/sites/default/files/allegati/ONS_2015_full.pdf	2015
Spain (Cancer Strategy of National Health System)	http://www.msps.es/organizacion/sns/planCalidadSNS/pdf/ActualizacionEstrategiaCancer.pdf	2009
Cervical cancer		
United States (USPSTF)	https://www.uspreventiveservicestaskforce.org/Page/Document/UpdateSummaryFinal/cervical-cancer-screening	2012
United States (ACS)	https://www.cancer.org/cancer/cervical-cancer/prevention-and-early-detection/cervical-cancer-screening-guidelines.html	2016
United States (ACOG)	http://www.acog.org/Patients/FAQs/Cervical-Cancer-Screening	2016
Luxembourg (Ministry of Health)	https://plancancer.lu/about/depistage/cancer-du-col-de-luterus/depistage-actuel-cancer-du-col-de-luterus/	
Switzerland (League Against Cancer)	https://www.liguecancer.ch/prevenir-le-cancer/depistage-et-prevention-du-cancer/cancer-du-col-de-luterus/prevention-et-depistage-du-cancer-du-col-de-luterus/	2010
Norway (Cancer Registry of Norway)	https://www.kreftregisteret.no/en/screening/Cervical-Cancer-Screening-Programme/ https://www.kreftregisteret.no/globalassets/masseundersokelsen-mot-livmorhalskreft/algoritme-hpv-test-i-primarscreening-ny-april-2015.jpg	2010
Netherlands (NIPHE)	http://www.rivm.nl/en/Topics/C/Cervical_cancer_screening_programme	2015
Germany (Federal Joint Committee)	https://www.g-ba.de/institution/themenschwerpunkte/frueherkennung/krebsfrueherkennung/	2015
Sweden (National Board of Health and Welfare)	http://www.socialstyrelsen.se/riktlinjer/nationellascreeningprogram/livmoderhalscancer-screeningme	2014
Ireland (National Screening Service)	http://www.screeningservice.ie/cervical.html	2009
Austria (Austrian Cancer Aid Society)	https://www.krebshilfe.net/information/krebs-vorsorge/frauen/	NR
Denmark (National Board of Health)	https://www.sst.dk/da/udgivelser/2014/~/media/31CB15B2A39E4A3EB198B9486F66B4A2.ashx	2014
Belgium (Foundation Against Cancer)	http://www.cancer.be/les-cancers-types-de-cancers-liste-z-cancer-du-col-de-lut-rus/examens	2017
Canada (CTFPHC)	http://canadiantaskforce.ca/guidelines/published-guidelines/cervical-cancer/	2013
Australia (Australian Government Department of Health)	http://www.cancerscreening.gov.au/internet/screening/publishing.nsf/Content/cervical-screening-1	2017
France (National Cancer Institute)	http://www.e-cancer.fr/Professionnels-de-sante/Depistage-et-detection-precoce/Depistage-du-cancer-du-col-de-l-uterus/Le-depistage-par-frottis-cervico-uterin	2017
Japan (National Cancer Center)	http://canscreen.ncc.go.jp/guideline/shikyukeigan.html http://canscreen.ncc.go.jp/guideline/shikyukeigan_eng.pdf	2010
Iceland (Icelandic Cancer Society)	https://www.krabb.is/leitarstod/um-leitarstodina/af-hverju-leghalskrabbameinsleit/spurningar-og-svor-um-leghalskrabbameinsleit-1?CacheRefresh=1 https://www.krabb.is/english/	NR
UK (UK National Screening Committee)	https://legacyscreening.phe.org.uk/cervicalcancer http://www.nhs.uk/conditions/Cervical-screening-test/Pages/Introduction.aspx	2016
Finland (Cancer Society of Finland)	https://www.cancer.fi/syoparekisteri/en/mass-screening-registry/cervical_cancer_screening/screening_programme/ https://www.cancer.fi/syoparekisteri/en/mass-screening-registry/cervical_cancer_screening/screening_programme/new_screening_technologies/	2010
New Zealand (Ministry of Health)	https://www.nsu.govt.nz/national-cervical-screening-programme/about-cervical-screening-programme	2014
Italy (National Screening Observatory)	http://www.osservatorionazionalescreening.it/sites/default/files/allegati/ONS_2015_full.pdf	2015
Spain (Cancer Strategy of National Health System)	http://www.msps.es/organizacion/sns/planCalidadSNS/pdf/ActualizacionEstrategiaCancer.pdf	2009
Colorectal cancer		
Australia (Australian Government Department of Health)	http://www.cancerscreening.gov.au/internet/screening/publishing.nsf/Content/bowel-screening-1	2016
Austria (Austrian Cancer Care)	https://www.krebshilfe.net/information/krebs-vorsorge/frauen/	N/A
Belgium (Foundation Against Cancer)	http://www.cancer.be/les-cancers/types-de-cancers/cancer-du-gros-intestin-colorectal/examens	2017
Canada (Canadian Task Force on Preventive Health Care)	https://canadiantaskforce.ca/guidelines/published-guidelines/colorectal-cancer/	2016
Denmark (National Board of Health)	https://www.sst.dk/da/udgivelser/2014/~/media/31CB15B2A39E4A3EB198B9486F66B4A2.ashx	2014
Finland (Cancer Society of Finland)	https://www.cancer.fi/@Bin/43261157/Sy%C3%B6p%C3%A4_Esite_Eng_Nettiin_080701.pdf	2010
France (Institut National du Cancer)	http://www.e-cancer.fr/Professionnels-de-sante/Depistage-et-detection-precoce/Depistage-du-cancer-colorectal/Le-programme-de-depistage-organise	2015
Germany (Federal Joint Committee)	https://www.g-ba.de/institution/themenschwerpunkte/frueherkennung/ueberblick/	2017
Iceland (Icelandic Cancer Society)	https://www.krabb.is/media/milliforsida/RISTILKRABBAMEIN-Sunna-Gudlaugsdottir.pdf	2015
Ireland (National Screening Service)	http://www.screeningservice.ie/bowel-screening.html	2012
Italy (National Screening Observatory)	https://www.osservatorionazionalescreening.it/sites/default/files/allegati/ONS_2015_full.pdf	2015
Japan (National Cancer Center)	http://canscreen.ncc.go.jp/guideline/daicyougan.html	2016
Luxembourg (Ministry of Health)	https://plancancer.lu/about/depistage/cancer-colorectal/objectifs-plan-cancer/	2016
Netherlands (National Institute for Public Health and the Environment)	http://www.rivm.nl/Onderwerpen/B/Bevolkingsonderzoek_darmkanker	2014
New Zealand (Ministry of Health)	http://www.health.govt.nz/our-work/diseases-and-conditions/cancer-programme/bowel-cancer-programme/national-bowel-screening-programme	2017
Norway (Cancer Registry of Norway)	https://www.kreftregisteret.no/en/screening/Screening-for-colorectal-cancer/	2012
Spain (Cancer Strategy of National Health System)	http://www.msps.es/organizacion/sns/planCalidadSNS/pdf/ActualizacionEstrategiaCancer.pdf	2009
Sweden (National Board of Health and Welfare)	http://www.socialstyrelsen.se/riktlinjer/nationellascreeningprogram/tjock-ochandtarmscancer-screen	2014
Switzerland (League against Cancer)	https://www.liguecancer.ch/prevenir-le-cancer/depistage-et-prevention-du-cancer/cancer-de-lintestin/programme-de-depistage-du-cancer-de-lintestin/	2013
United Kingdom (UK National Screening Committee)	https://www.gov.uk/government/uploads/system/uploads/attachment_data/file/538524/Screening_in_the_UK___making_effective_recommendations_2015_to_2016_180716_final.pdf	2016
United States (American Cancer Society)	https://www.cancer.org/cancer/colon-rectal-cancer/detection-diagnosis-staging/acs-recommendations.html	2017
United States (American College of Gastroenterology)	http://gi.org/wp-content/uploads/2017/06/ajg2017174a.pdf	2017
United States (U.S. Preventive Services Task Force)	https://www.uspreventiveservicestaskforce.org/Page/Document/RecommendationStatementFinal/colorectal-cancer-screening2#tab	2016
Prostate cancer		
Australia (Australian Government Department of Health)	http://www.cancerscreening.gov.au/internet/screening/publishing.nsf/Content/prostate-cancer-screening	2017
Austria (Austrian Cancer Care)	https://www.krebshilfe.net/information/krebs-vorsorge/maenner/	2015
Belgium (Foundation Against Cancer)	http://www.cancer.be/les-cancers/types-de-cancers/cancer-de-la-prostate/examens	2017
Canada (Canadian Task Force on Preventive Health Care)	https://canadiantaskforce.ca/guidelines/published-guidelines/prostate-cancer/	2014
Denmark (National Board of Health)	No available recommendations concerning prostate cancer	
Finland (Cancer Society of Finland)	No available recommendations concerning prostate cancer	
France (Institut National du Cancer)	http://www.e-cancer.fr/Professionnels-de-sante/Depistage-et-detection-precoce/Depistage-du-cancer-de-la-prostate	2016
Germany (Federal Joint Committee)	https://www.g-ba.de/institution/themenschwerpunkte/frueherkennung/ueberblick/	2017
Iceland (Icelandic Cancer Society)	https://www.krabb.is/fraedsla-forvarnir/krabbamein-a-o/blodruhalskirtilskrabbamein/	2016
Ireland (National Screening Service)	No available recommendations concerning prostate cancer	
Italy (National Screening Observatory)	No available recommendations concerning prostate cancer	
Japan (National Cancer Center)	http://canscreen.ncc.go.jp/guideline/zenritsusengan.html	2009
Luxembourg (Ministry of Health)	https://plancancer.lu/about/depistage/cancer-de-la-prostate/	2014
Netherlands (National Institute for Public Health and the Environment)	No available recommendations concerning prostate cancer	
New Zealand (Ministry of Health)	http://www.health.govt.nz/our-work/diseases-and-conditions/cancer-programme/prostate-cancer-programme	2016
Norway (Cancer Registry of Norway)	No available recommendations concerning prostate cancer	
Spain (Cancer Strategy of National Health System)	http://www.msps.es/organizacion/sns/planCalidadSNS/pdf/ActualizacionEstrategiaCancer.pdf	2009
Sweden (National Board of Health and Welfare)	http://www.socialstyrelsen.se/riktlinjer/nationellascreeningprogram/prostatacancer-screeningmedpsa	2016
Switzerland (League against Cancer)	https://www.liguecancer.ch/prevenir-le-cancer/depistage-et-prevention-du-cancer/depistage-du-cancer-de-la-prostate/	2015
United Kingdom (UK National Screening Committee)	https://www.gov.uk/government/uploads/system/uploads/attachment_data/file/538524/Screening_in_the_UK___making_effective_recommendations_2015_to_2016_180716_final.pdf	2016
United States (American Cancer Society)	https://www.cancer.org/cancer/prostate-cancer/early-detection/acs-recommendations.html	2016
United States (American Urological Association)	https://www.auanet.org/guidelines/early-detection-of-prostate-cancer-(2013-reviewed-and-validity-confirmed-2015)	2015
United States (U.S. Preventive Services Task Force)	https://www.uspreventiveservicestaskforce.org/Page/Document/UpdateSummaryFinal/prostate-cancer-screening	2012
Skin cancer		
United States (USPSTF)	https://www.uspreventiveservicestaskforce.org/Page/Document/UpdateSummaryFinal/skin-cancer-screening2	2016
United States (ACS)	https://www.cancer.org/cancer/skin-cancer/prevention-and-early-detection/skin-exams.html	2016
Germany (Federal Joint Committee)	https://www.g-ba.de/institution/themenschwerpunkte/frueherkennung/krebsfrueherkennung/	2017
Austria (Austrian Cancer Aid Society)	https://www.krebshilfe.net/information/krebs-vorsorge/frauen/	NA
France (National Cancer Institute)	http://www.e-cancer.fr/Professionnels-de-sante/Depistage-et-detection-precoce/Detection-precoce-des-cancers-de-la-peau/Aide-pour-votre-pratique	2016
Lung cancer		
United States (USPSTF)	https://www.uspreventiveservicestaskforce.org/Page/Document/UpdateSummaryFinal/lung-cancer-screening	2013
United States (ACS)	https://www.cancer.org/health-care-professionals/american-cancer-society-prevention-early-detection-guidelines/lung-cancer-screening-guidelines.html	2013
Canada (CTFPHC)	http://canadiantaskforce.ca/guidelines/published-guidelines/lung-cancer/	2016
Australia (Australian Government Department of Health)	http://www.cancerscreening.gov.au/internet/screening/publishing.nsf/Content/lung-cancer-screening	2015
Japan (National Cancer Center)	http://canscreen.ncc.go.jp/guideline/haigan.html	NA
UK (UK National Screening Committee)	https://legacyscreening.phe.org.uk/lungcancer	2006
